# Amygdala reactivity in ethnic minorities and its relationship to the social environment: an fMRI study

**DOI:** 10.1017/S0033291717003506

**Published:** 2018-01-12

**Authors:** Robert McCutcheon, Michael A. P. Bloomfield, Tarik Dahoun, Marina Quinlan, Sylvia Terbeck, Mitul Mehta, Oliver Howes

**Affiliations:** 1 Department of Psychosis Studies, Institute of Psychiatry, Psychology & Neuroscience, Kings College London, De Crespigny Park, London SE5 8AF, UK; 2 Psychiatric Imaging Group, Robert Steiner MR Unit, MRC London Institute of Medical Sciences, Hammersmith Hospital, London W12 0NN, UK; 3 Faculty of Medicine, Psychiatric Imaging Group, Institute of Clinical Sciences, Imperial College London, Du Cane Road, London W12 0NN, UK; 4 Division of Psychiatry, University College London, 6th Floor, Maple House, 149 Tottenham Court Road, London WC1T 7NF, UK; 5 Clinical Psychopharmacology Unit, Research Department of Clinical, Educational and Health Psychology, University College London, 1–19 Torrington Place, London WC1E 6BT, UK; 6 School of Psychology, Plymouth University, Drake Circus, Plymouth PL48AA, UK; 7 Department of Neuroimaging, Institute of Psychiatry, Psychology & Neuroscience, Kings College London, De Crespigny Park, London SE5 8AF, UK

**Keywords:** Amygdala, ethnic density, ethnicity, ethnic minority, fMRI, paranoia, psychosis, risk factors, schizophrenia

## Abstract

**Background:**

Ethnic minority individuals have an increased risk of developing a psychotic disorder, particularly if they live in areas of ethnic segregation, or low own group ethnic density. The neurobiological mechanisms underlying this ethnic minority associated risk are unknown. We used functional MRI to investigate neural responses to faces of different ethnicity, in individuals of black ethnicity, and a control group of white British ethnicity individuals.

**Methods:**

In total 20 individuals of black ethnicity, and 22 individuals of white British ethnicity underwent a 3T MRI scan while viewing faces of black and white ethnicity. Own group ethnic density was calculated from the 2011 census. Neighbourhood segregation was quantified using the Index of Dissimilarity method.

**Results:**

At the within-group level, both groups showed greater right amygdala activation to outgroup faces. Between groups, the black ethnicity group showed greater right amygdala activation to white faces, compared to the white ethnicity group. Within the black ethnicity group, individuals living in areas of lower own group ethnic density showed greater right amygdala reactivity to white faces (*r* = −0.61, *p* = 0.01).

**Conclusions:**

This is the first time an increased amygdala response to white faces has been demonstrated in individuals of black ethnicity. In the black ethnicity group, correlations were observed between amygdala response and neighbourhood variables associated with increased psychosis risk. These results may have relevance for our understanding of the increased rates of paranoia and psychotic disorders in ethnic minority individuals.

## Background

Being a member of an ethnic minority group is one of the most well established environmental risk factors for schizophrenia (Cantor-graae *et al.* 2005; Bourque *et al.* 2011). Early research focused on the risk associated with migration (Ødegaard, 1932). It has subsequently become clear that this increased risk is not secondary to selective migration (Selten *et al.* 2002; van der Ven *et al.* 2015), and the effect persists among second-generation migrants, indicating that the risk is associated with being a member of a minority group, rather than the act of migration itself (Hutchinson *et al.* 1996; Bresnahan *et al.* 2007; Bourque *et al.* 2011). In various settings, the increased risk appears greatest for individuals of black ethnicity (Cantor-graae *et al.* 2005; Bresnahan *et al.* 2007). In the UK, individuals of black ethnicity were found to have incidence rate ratios compared with the white British population of between six and nine (Fearon *et al.* 2006). This risk also appears to be somewhat specific for psychosis. Although there is some evidence that affective disorders may have slightly increased incidence rates in first-generation immigrants, this appears to be driven by manic presentations, and there is no evidence of increased rates of depression or anxiety in the UK black ethnicity population (Shaw *et al.* 1999; Brugha *et al.* 2004; Weich *et al.* 2004; Swinnen & Selten, 2007; Mindlis & Boffetta, 2017). Similarly, in the USA a number of studies have reported reduced rates of depression and anxiety in African Americans (Kessler *et al.* 2003; Riolo *et al.* 2005; Breslau *et al.* 2006; Himle *et al.* 2009; Asnaani *et al.* 2010; Gibbs *et al.* 2013).

Interestingly, despite the fact that communities with high proportions of ethnic minority individuals are frequently the most deprived, living in an area where one's own ethnic group forms a larger proportion of the population is relatively protective against schizophrenia (Shaw *et al.* 2012; Kirkbride *et al.* 2014). One hypothesis is that higher own group ethnic density buffers the individual from the adverse psychiatric consequences of social isolation and racist experiences (Bécares *et al.* 2009). Epidemiological work has also suggested that greater levels of segregation between members of ethnic minority groups and the rest of the community may be linked to a greater incidence of psychotic disorders (Kirkbride *et al.* 2014).

Cognitive models propose that minority status is associated with greater levels of social threat (Combs *et al.* 2002; Morgan *et al.* 2010). However, the neurobiological mechanisms underlying both the increased risk of psychosis in ethnic minority groups, and the protective effects of increased own group ethnic density, are not known. One previous fMRI study investigated the neural correlates of the stress response in Turkish migrants living in Germany, and identified differences in anterior cingulate activation (Akdeniz *et al.* 2014). The amygdala is also an area of potential interest given it is a key component of the neural circuit processing threatening experiences (Chekroud *et al.* 2014; Fox *et al.* 2015; Underwood *et al.* 2016).

A greater right amygdala neural response to pictures of faces of people with an outgroup ethnicity, compared with those with in-group ethnicity, has been interpreted as representing threat appraisal (Chekroud *et al.* 2014). This outgroup effect has been consistently demonstrated in multiple studies of individuals of white ethnicity, but has not been thoroughly investigated in individuals of black ethnicity (Cunningham *et al.* 2004; Lieberman *et al.* 2005; Wheeler & Fiske, 2005; Ronquillo *et al.* 2007; Chekroud *et al.* 2014; Fox *et al.* 2015). In psychosis, meanwhile, amygdala hyperactivity has been linked to paranoid symptoms (Goghari *et al.* 2010; Pinkham *et al.* 2015; Underwood *et al.* 2016). Individuals from ethnic minority populations typically have much greater exposure to outgroup individuals than the native population. Amygdala hyperactivity to frequent outgroup exposure could therefore partially underlie the greater levels of paranoia seen in both ethnic minority patient and population samples (Bhugra *et al.* 2000; Combs *et al.* 2002; Cohen *et al.* 2004; Veling *et al.* 2007; Wickham *et al.* 2014), and potentially have relevance as regards the increased rates of psychotic illnesses in these groups.

In view of this evidence, we hypothesised that individuals who identified as being of black ethnicity would display a greater right amygdala response to white faces, than individuals who identified as being of white ethnicity; who would, in turn, show a greater response to black faces. Within the black ethnic minority group, we further hypothesised that increased amygdala reactivity to white faces would correlate with increased ethnic segregation, and decreased own group ethnic density, as these ethnicity related neighbourhood variables are associated with increased psychosis risk.

## Methods

### Participants

Healthy volunteers were recruited throughout the UK via newspaper, online and leaflet advertising, and in person recruitment, as part of a study examining environmental risk factors for psychosis. Participants were aged 18–45, with no history of mental illness, in good physical health, and with the capacity to give informed consent.

The black ethnic minority group needed to be a first or second generation migrant, to self-identify as being of black ethnicity, and were classified as of either black Caribbean or black African ethnicity based on either their own (first generation migrants) or their parents’ (second generation migrants) country of origin. The white British ethnicity group needed to self-identify as being of white British ethnicity and have at least one parent of UK nationality. The white British ethnicity group was age-matched within 5 years to the black ethnic minority group. All subjects provided written informed consent after the study had been fully explained.

### Sociodemographic assessment

Population density, and own group ethnic density as a fraction of the total population were obtained from the 2011 census; Indices of Multiple Deprivation (where a lower ranking equates to a more deprived area) were obtained from 2015 data (English Indices of Deprivation, 2015). Values were obtained for each participant's current statistical lower layer super output area (LSOA, geographical areas with populations of roughly 1500 individuals).

In order to quantify ethnic segregation, the Index of Dissimilarity method was used to calculate an index of segregation at the ward-level, using census output areas as the smaller geographic unit. A score of 0 is equivalent to no segregation, while a score of 1 is equivalent to complete segregation (see online Supplementary Methods for further information) (Yalonetzky, 2015). Own group ethnic density and ethnic segregation were both calculated with participants assigned to either black African, black Caribbean or white British ethnicity.

### fMRI task

**Fig. 1.**
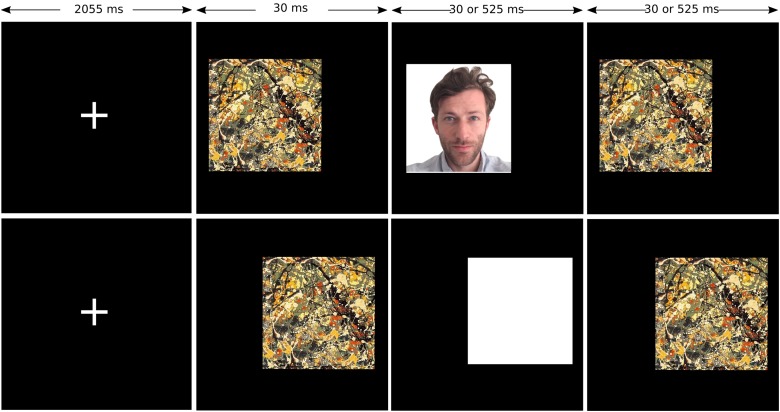
fMRI task. Participants judge whether an image is to the left or right of a fixation cross. Each face trial (top row) is followed by four white square trials (bottom row).

An event-related, implicit face perception task was employed based on a previously reported experimental design (see Fig. 1) (Cunningham *et al.* 2004). During the task, participants indicated whether a visual stimulus appeared to the left or right of a fixation cross. The stimuli consisted of either black or white male faces presented for 30 ms (short trials) or 525 ms (long trials) or a white square also presented for 30 or 525 ms. Faces were taken from the Stanford University faces database (https://stanforduniversity.qualtrics.com/SE/?SID=SV_aX0ovSkASZR9Py4), 18 faces of each ethnicity were chosen and matched on ratings of attractiveness, stereotypicality and age. In order to mask the short duration stimuli, all stimuli were preceded by an abstract picture for 30 ms and followed by the same picture for either 525 ms (short trials) or 30 ms (long trials). As a result, all trials lasted a total of 585 ms. During the short trials, the participant's experience was of seeing an abstract picture and they were not aware of the implicitly presented face. We checked this after the experiment and no participant reported seeing a face during the presentation of these stimuli. During the long trials, they saw either a white square or a face. A fixation cross appeared for 2055 ms between trials. Each face trial was followed by four white square trials giving an interstimulus interval between faces ranging from 10.62 to 11.12 s. Participants completed three runs. During each run participants would be presented with six short trials of black faces, six long trials of black faces, six short trials of white faces and six long trials of white faces, shown in a random order without replacement.

### Data acquisition

Imaging data were acquired using a Philips 3T Intera magnetic resonance imaging system. Functional imaging consisted of T2* weighted transverse echo planar image (EPI) slices. Each run consisted of 161 volumes, collected in an ascending direction, with 2.19 mm × 2.19 mm voxel dimensions in the plane, repetition time (TR) – 2000 ms, and echo time (TE) –30 ms.

The EPI scans were followed by a gradient-echo scan (TR = 9.7 ms, TE = 4.6 ms, flip angle –9°, slice thickness 1.20 mm, 0.94 × 0.94 mm^2^ in-plane resolution, 150 slices).

### fMRI data analysis

Analysis of fMRI data was undertaken using SPM8 (http://www.fil.ion.ucl.ac.uk/spm/) in Matlab 7.9. The first two volumes for each participant were discarded so as to avoid T1 equilibration effects. Slice timing correction was applied to each volume. Spatial realignment to the third volume was followed by coregistration of each participant's functional and anatomical data. Images were then normalised into standard MNI space using the normalization parameters estimated by T1 structural image unified segmentation. Smoothing of the resampled images (3 mm × 3 mm × 3 mm) was with a Gaussian kernel of 8 mm full-width-half-maximum.

First level analysis using the general linear model included six conditions (short and long presentations of the white square, white faces, and black faces), and individual events were convolved with the canonical haemodynamic response function. The model included the six movement parameters from realignment, and six-volume to volume movement parameters, as regressors of no interest, additionally any volumes where between volume movement was greater than 0.5 mm were removed. We excluded any runs with extended shifts of over 2 mm. Participants were included in the second level analysis if they had at least two usable runs. First level contrasts were constructed between faces and white squares.

The second level within-group analyses were performed using paired *t* tests. The second level between groups analyses were performed using independent samples *t* tests, and a 2 × 2 flexible factorial ANOVA implemented in SPM8. Following from a priori hypothesis, region of interest (ROI) analyses were conducted for the amygdala using Marsbar (http://marsbar.sourceforge.net/) and the included Automated Anatomical Labelling ROI library. As per SPM conventions *t* test *p* values are reported as one-tailed.

### Statistical analysis

Analyses were carried out using SPSS for Macintosh version 23.0. After exclusion of outliers of more than 1.5 interquartile ranges below or above the 1st or 3rd quartiles, continuous variables were assessed for normality using the Shapiro–Wilks test. Differences between group means were assessed using an independent samples *t* test for normally distributed variables, after using Levene's test to check for equality of variances. Mann–Whitney *U* tests were used for non-normally distributed variables. Between-group differences in terms of the magnitude of the amygdala outgroup effect, was tested with a two-tailed independent samples *t* test. Correlations between normally distributed variables were assessed using Pearson's product moment coefficient, and were only reported if they remained significant after removal of outliers defined as a Cook's *d* of >*n*/4. Correlations involving non-normally distributed variables were assessed using Spearman's rank correlation coefficient. All correlations were two-tailed with *p* < 0.05 defined as significant. Neighbourhood variables found on bivariate testing to show a significant correlation with right amygdala response were subsequently entered into a stepwise regression with right amygdala response as the dependent variable.

### Data availability

Imaging and demographic data are available from the corresponding author on request.

## Results

### Sociodemographic and neighbourhood variables

A total of 42 individuals were included in the study (black ethnic minority group *n* = 20, white British ethnicity group *n* = 22). There were no significant differences between groups in terms of sex or age. In terms of neighbourhood variables the black ethnic minority group had significantly lower own group ethnic density percentages and lived in significantly more deprived, less segregated, and more densely populated areas (demographics of the entire sample are reported in online Supplementary Table S1, while details of those included in the imaging analysis are described in Table 1). In the black ethnicity group, participant characteristics in terms of age, gender, and deprivation were similar to UK averages. In the white ethnicity group, the study population was younger compared with the UK median age but otherwise similar (see online Supplementary Material).

**Table 1.** Demographic characteristics of participants included in imaging analysis

	Black ethnic minority (*n* = 17)	White British ethnicity (*n* = 19)	*P*
Age (years), median (IQR)	24.5 (20.8–32.2)	24.0 (22.5–28.1)	0.98^a^
Sex *n* (%)			
Male	7 (41)	9 (50)	0.71^b^
Female	10 (59)	10 (53)	
Migration *n* (%)			
1st generation	7 (41)		
2nd generation	10 (59)		
Black Caribbean, *n* (%)	3 (18)		
Black African, *n* (%)	14 (82)		
% own ethnicity, median (IQR)	9.1 (6.0–24.6)	86.3 (77.2–95.4)	<0.001^a^
Index of multiple deprivation rank/1000, median (IQR)	7.2 (5.0–13.5)	22.8 (19.0–31.4)	<0.001^a^
Index of segregation, median (IQR)	0.28 (0.18–0.31)	0.32 (0.28–0.42)	0.008^a^
Population density: persons per hectare, median (IQR)	91.7 (59.1–126.7)	38.6 (1.9–96.1)	0.005^a^

a Mann–Whitney.

b χ^2^ test.

Within the black ethnic minority group, own group ethnic density negatively correlated with the degree of segregation (*r*_*s*_ = −0.809, *p* < 0.001) (correlations between other neighbourhood variables are described in the online Supplementary Data).

### Functional imaging results

Three participants from each group were excluded due to excessive movement or failed scans. For the 30ms presentations neither black nor white faces elicited significant right amygdala activation in either the white British or black ethnic minority groups (all contrasts against the white square baseline *p* > 0.05). In addition, there were no significant differences between groups, or within groups between stimuli, for the 30 ms presentations. All results reported below are for 525 ms presentations.

**Fig. 2.**
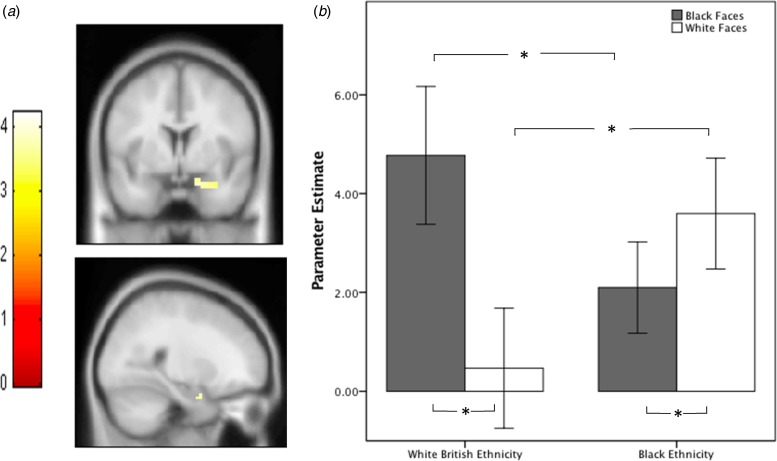
(a) Increased amygdala activation in black ethnicity group compared with white British ethnicity group for white > black faces contrast during 525 ms presentations. Functional maps are unmasked and thresholded at *p* < 0.001 whole brain, uncorrected for display purposes. (b) Mean parameter estimates for right amygdala region of interest (ROI) for both groups, for faces (525 ms) *v.* white square contrasts, error bars = ±SEM. *p < 0.05.

At the within-group level, both groups individually showed greater right amygdala activation to outgroup faces (white British ethnicity group *t* = 1.90, *p* = 0.036; black ethnic minority group *t* = 2.38, *p* = 0.015) (see Fig. 2). This was reflected in a significant group × task interaction (*F* = 7.85, *p* = 0.008) (see Fig. 2). The magnitude of the outgroup effect was not significantly different between groups (*t* = 1.1, *p* = 0.3).

Between groups, compared with the white British ethnicity group, the black ethnic minority group showed significantly greater right amygdala activation to white faces (*t* = 1.84, *p* = 0.038), and significantly lower right amygdala activation to black faces (*t* = 1.80, *p* = 0.040) (see Fig. 2).

Whole brain exploratory analyses were conducted to investigate the potential involvement of other regions in the effects and to enable comparisons with studies investigating other regions (peak threshold *p* < 0.001, cluster threshold *p* < 0.05 FWE corrected). No significant results were observed for the between groups, or group × task interaction analyses. For the black ethnicity group, white faces > black faces contrast one small cluster was observed in the right cerebellum (*p* = 0.007, cluster size −76 voxels, coordinates *x* = 33, *y* = −58, *z* = −26).

### Relationships between imaging results and neighbourhood variables

**Fig. 3.**
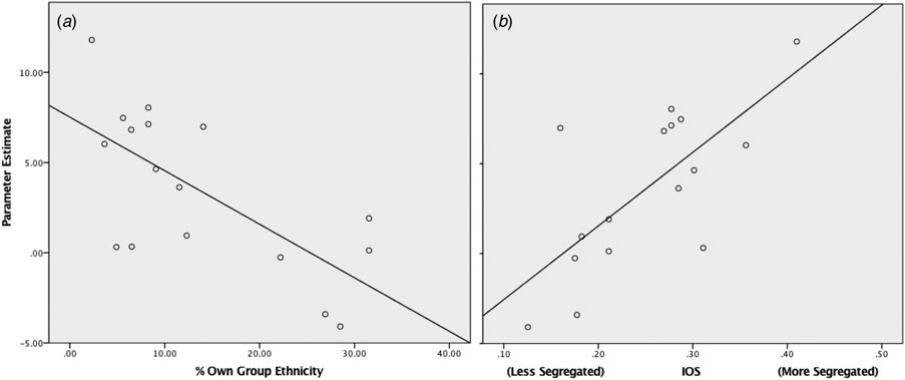
Scatter plots for right amygdala response to whitefaces within the black ethnicities group, against: (a) % own group ethnicity of current lower layer super output area (LSOA) (*r*_s_ = −0.611, *p* = 0.009) (b) Index of segregation (*r*_*p*_ = 0.831, *p* < 0.001).

Within the black ethnic minority group right amygdala response to white faces significantly correlated with living in an area of greater ethnic segregation (*r*_*p*_ = 0.831, *p* < 0.001) and lower own group ethnic density (*r*_*s*_ = −0.611, *p* = 0.009) (see Fig. 3 and online Supplementary Material for results using alternative measures of ethnic density). The right amygdala response also positively correlated with greater population density (*r*_*p*_ = 0.627, *p* = 0.002) and lower levels of deprivation (*r*_*s*_ = 0.601, *p* = 0.011). These findings survive Bonferroni correction accounting for the four separate tests (*p* < 0.0125). All four neighbourhood variables were entered into a stepwise regression with right amygdala response as the dependent variable; from this only own group ethnic density remained a significant predictor (*r* = 0.689, *p* = 0.002, see Table 2). In the white ethnicity group, no significant correlations were observed between neighbourhood variables and right amygdala response to black faces.

**Table 2.** Linear regression of relationship between of neighbourhood variables and right amygdala reactivity to white faces

Variable	*B*	SE	Beta	*T*	*P*	*R*^2^	*P*
Model A							
Constant	3.44	6.41		0.537	0.601	0.571	0.027
Own group ethnic density	−0.172	0.164	−0.399	−1.05	0.315		
Index of segregation	−12.5	12.5	−0.283	−0.996	0.339		
Own group ethnic density	0.032	0.029	0.345	1.11	0.288		
Index of multiple deprivation	<0.001	<0.001	0.363	1.36	0.199		
Model B							
Constant	7.52	1.37		5.51	<0.001	0.475	0.002
Own group ethnic density	−0.297	0.081	−0.689	−3.68	0.002		

Model A includes all neighbourhood variables. Model B includes only ‘own group ethnicity’ as determined by a stepwise regression.

## Discussion

We demonstrated increased amygdala activation to white faces in a group of individuals of black ethnicity – both within group compared with black faces and between groups when compared with white British individuals. The white British ethnicity group showed a greater amygdala response to black faces both within, and between the group. In addition, within the black ethnic minority group, a stepwise regression including neighbourhood variables that correlated with amygdala reactivity, found that lower own group ethnic density was significantly associated with greater amygdala reactivity to white faces.

Our finding of increased right amygdala reactivity to black faces in the white British ethnicity group has been repeatedly demonstrated in white ethnicity participants (Cunningham *et al.* 2004; Lieberman *et al.* 2005; Wheeler & Fiske, 2005; Ronquillo *et al.* 2007; Chekroud *et al.* 2014). This is, however, to our knowledge the first time that increased reactivity to white faces has been unambiguously demonstrated in individuals of black ethnicity. One previous study demonstrating greater amygdala activity to outgroup faces included four participants of black ethnicity but did not comment on this subgroup specifically (Hart *et al.* 2000). A study by Lieberman *et al.* found that African-American ethnic minority individuals showed an increased amygdala response while viewing black faces (Lieberman *et al.* 2005). This study, however, included only nine African-American subjects and used a different task that focused on differences in perceptual and verbal encoding, which may explain the inconsistency with our findings. While our findings suggest a general out-group effect, this finding may potentially have a greater psychological impact for ethnic minority groups given their generally greater exposure to outgroups.

This is also the first time that amygdala reactivity has been linked to neighbourhood variables related to ethnic minority associated risk for psychosis. Living in areas of low own group ethnic density has been shown to increase the risk of psychosis in ethnic minorities (Bosqui *et al.* 2014). Our results extend these findings by indicating that lower own group ethnic density is also linked to greater amygdala response to outgroup faces in black individuals. Living in an environment where one is more isolated from one's own ethnic group (i.e. an area of low own group ethnic density), could conceivably contribute to outgroup individuals being perceived as a greater threat. Taken with evidence that experiences of racism are correlated with amygdala activation to white faces in black individuals (Greer *et al.* 2012), this suggests that environmental exposures are associated with functional alterations in the brain circuits involved in threat processing. While a number of our results were significant when examining the bilateral amygdala (see online Supplementary Material), our findings were driven by the right amygdala. The right amygdala appears to be particularly implicated in the processing of race, although the underlying reasons for this remain unclear (Chekroud *et al.* 2014).

### Methodological considerations

We investigated a group of black ethnic minority individuals who were first or second generation migrants relative to the white British ethnic majority. This group appears to display the greatest increase in the incidence of psychosis, compared with white British individuals (Fearon *et al.* 2006; Coid *et al.* 2008). However, it should be recognised that other ethnic groups, including non-British white individuals, and Asian individuals, have increased rates of psychosis as well, and migrants may be exposed to other adverse experiences in addition to those associated with being a member of a minority group (Fearon *et al.* 2006; Coid *et al.* 2008). Ethnic minority status itself is likely a crude proxy for multiple complex, interacting factors that cumulatively act to increase the risk of psychosis. Our study used a convenience sampling procedure and future work should determine how representative the sample is of the population in general.

Our finding of an association between ethnicity related neighbourhood variables and amygdala reactivity suggests that this mechanism may have some relevance for our understanding of the neurobiological basis of ethnic minority associated psychosis risk. This, however, is a speculative interpretation, and the current study does not establish causality. Capturing the relationship between amygdala functioning and real-world experiences of being in a minority group could potentially be accomplished using experience sampling methodologies (Gevonden *et al.* 2016; Reininghaus *et al.* 2016).

Study participants were intentionally unaffected by mental illness, which precluded investigating direct associations with psychopathology. As our participants did not have a mental illness it is important to recognise the possibility that our findings may be interpreted as physiologically appropriate or neurobiological correlates of psychological resilience. However, the fact that in patients with schizophrenia greater amygdala responses are associated with paranoid symptoms suggests this may not be the case (Goghari *et al.* 2010; Pinkham *et al.* 2015; Underwood *et al.* 2016). Amygdala dysfunction is implicated in a wide range of disorders, so even if aberrations identified in the current study have pathoaetiological relevance, it may not be specific to psychotic disorders. Furthermore, drawing parallels with patient groups is limited by the fact that our task specifically measured differences in amygdala reactivity to faces of differing ethnicities, whereas most probes used in patient populations tend to involve emotion processing. Investigating amygdala response to faces of differing ethnicity in patient populations would assist with determining if this mechanism is linked to psychopathology.

When relating neighbourhood variables to amygdala response we were primarily interested in the influence of own group ethnic density, and ethnic segregation. We additionally measured population density and deprivation ranking. The stepwise regression resulted in a model only including own group ethnic density. Nearly all these variables, however, are strongly correlated with one another and thus determining which play mediating as opposed to confounding roles is not possible in the current study. Larger sample sizes would allow for the investigation of interactions between variables, while replication in other settings would establish whether the degree to which the effect is generalizable. Additionally, while the link between psychosis risk and own group ethnic density is well established, the evidence for the influence of ethnic segregation is less clear (Kirkbride *et al.* 2007, 2014).

We did not observe significant between or within group differences, for the 30 ms stimuli presentations. This is surprising given that this subliminal presentation has previously been found to evoke greater amygdala responses to outgroup faces than the 525 ms presentation (Cunningham *et al.* 2004). Potential reasons underlying this discrepancy may include differences in masking stimuli between the current and previous study, or differences as regards image characteristics such as levels of brightness and contrast which subliminal stimuli may be particularly sensitive to.

### Implications for understanding the mechanisms underlying ethnic minority associated risk for psychosis

Cognitive models of psychosis propose an increased level of social threat anticipation as part of a pathway to the development of persecutory beliefs (Bentall & Fernyhough, 2008). Of note, a higher degree of paranoid symptomatology has been described in ethnic minority groups, both in patient (Bhugra *et al.* 2000; Veling *et al.* 2007), and healthy populations (Combs *et al.* 2002; Cohen *et al.* 2004; Wickham *et al.* 2014). In the current study, both the black ethnic minority group and the white British ethnicity group displayed increased amygdala reactivity to outgroup faces. The consequence for each group, however, may be different given that ethnic minorities typically have much greater outgroup contact in day-to-day life. Following from this, if the increased amygdala response to outgroup faces is interpreted as a marker of threat, this suggests that ethnic minority individuals have more frequent threat experience.

Although amygdala hyperactivity has been linked to paranoid symptoms in psychosis (Goghari *et al.* 2010; Pinkham *et al.* 2015; Underwood *et al.* 2016), reactivity during facial processing is not consistently raised in schizophrenia (Taylor *et al.* 2012). The black ethnic minority group in the current study did not show overall increased amygdala reactivity, only to specific stimuli. A differential sensitivity to various stimuli may partially explain the variability of findings observed in studies of amygdala function in schizophrenia.

## Conclusions

Amygdala reactivity to white faces is increased in black ethnic minority individuals, and correlated with measures of own group ethnic density and segregation. These findings indicate that black ethnic minority individuals show the same response to outgroup faces seen in white ethnic majority groups, and also suggest that in this population ethnic segregation and lower own group ethnic density is associated with greater amygdala reactivity to outgroup faces.

## Supplementary material

For supplementary material accompanying this paper visit http://dx.doi.org/10.1017/S0033291717003506. click here to view supplementary material
