# Supplementary material for: Amygdala reactivity in ethnic minorities and its relationship to the social environment: an fMRI study
Source: Psychol Med. 2018 Jan 12;48(12):1985–92. doi: 10.1017/S0033291717003506 (PMC5933521; doi:10.1017/S0033291717003506)
Supplement: Supplementary file 1 [file S0033291717003506sup001.docx]

**Supplementary information**

**Methods**

*Ethnic classification*

Individuals were assigned to the white British or black ethnic minority group according to their self-identified ethnic group. Within the black ethnic minority group, individuals were classified as of either black Caribbean or black African ethnicity based on either their own (first generation migrants), or their parents’ (second generation migrants) country of origin.

When calculating own group ethnic densities and indices of dissimilarity neighborhood proportions were calculated from the most recent (2011) census as follows:

A)The black African population in a neighborhood was defined as the sum of both: i)Mixed/multiple ethnic group: White and Black African & Ii)Black/African/Caribbean/Black British: African

B)The black Caribbean population in a neighborhood was defined as the sum of both: i)Mixed/multiple ethnic group: White and Black Caribbean & ii)Black/African/Caribbean/Black British: Caribbean

C)The white population in a neighborhood was defined as those in the census category: White: English/Welsh/Scottish/Northern Irish/British, Irish, Gypsy or Irish Traveller, Other White

*Ethnic Density*

For both groups this was calculated as the proportion of one’s own ethnic group (African, Caribbean or white; as defined above) as a percentage of the Lower Super Output Area’s (LSOA) total population.

*Index of dissimilarity*

For ethnic minority individuals the index of dissimilarity (IOD) was calculated as regards to their own ethnic group (i.e. African or Caribbean) in relation to the white population. IODs were calculated for the ward rather than LSOA level, as LSOAs are smaller and therefore do not contain a sufficient number of output areas for a meaningful calculation. The IOD was calculated as (in this example for Black African participants):

$$\mathrm{IOD}=\frac{1}{2}\sum_{i=1}^{n} \left| \frac{{Woa}_{i}}{Wward}- \frac{{BAoa}_{i}}{BAward} \right|$$

*n= number of output areas within the ward*

*BAoa_i_= Number of black African individuals within output area i*

*BAward= Number of black Africans within the ward*

*Woa_i_= Number of white individuals within output area i*

*Wward= Number of white individuals within the ward*

The same approach was adopted for black Caribbean participants. For the white British ethnicity participants the IOD was calculated as regards the white population (as defined above) in relation to the sum of i) Black/African/Caribbean/Black British: African, Caribbean, Other Black AND ii) Mixed/multiple ethnic group: White and Black Caribbean, White and Black Caribbean.

***Statistical Analysis***

Power calculations were conducted with the software package G*Power. For α≤0.05 and β≥0.8, a sample size of 20 for each group allows for within group correlations of r≥0.55 (two-tailed), and within group differences in mean activation of d=0.58 (cohen’s d, one-tailed) to be detected, and between group mean differences of d=0.8 to be detected (cohen’s d, one-tailed). Analyses were carried out using SPSS for Macintosh version 23.0. After exclusion of outliers of more than 1.5 interquartile ranges below or above the 1^st^ or 3^rd^ quartiles, continuous variables were assessed for normality using the Shapiro-Wilks test. Differences between group means were assessed using an independent samples *t*-test for normally distributed variables, after using Levene’s test to check for equality of variances. Mann-Whitney *U* tests were used for non-normally distributed variables. Correlations between normally distributed variables were assessed using Pearson’s product moment coefficient, and were only reported if they remained significant after removal of outliers defined as a Cook’s *d* of >n/4. Correlations involving non-normally distributed variables were assessed using Spearman’s rank correlation coefficient. All correlations were two-tailed with *p*<0.05 defined as significant.

**Results**

|  | Black Ethnic minority (n=20) | White British Ethnicity (n=22) | P |
| --- | --- | --- | --- |
| Age (years), median (IQR) | 25.1 (21.1-32.2) | 24.0 (22.5-28.5) | 0.67^a^ |
| Sex n(%): Male | 9 (45) | 11 (50) | 0.88^c^ |
| Female | 11(55) | 11 (50) |  |
| Migration n(%): 1^st^ generation | 7 (35) |  |  |
| 2^nd^ generation | 13(65) |  |  |
| Black Caribbean , n (%) | 4 (20) |  |  |
| Black African, n (%) | 16 (80) |  |  |
| % own ethnicity, median (IQR) | 10.3 (6.5-25.7) | 89.1 (77.8-95.7) | <0.001^b^ |
| Index of Multiple deprivation rank/1000, median (IQR) | 7.6 (4.8-10.6) | 20.9 (17.0-29.6) | <0.001^b^ |
| Index of segregation, median (IQR) | 0.27 (0.18-0.30) | 0.33 (0.28-0.43) | 0.004^b^ |
| Population density: persons per hectare, median (IQR | 92.1 (57.0-126.8) | 42.8 (1.8-83.6) | 0.003^b^ |

**Supplementary table 1:** Demographic characteristics of full sample participants (including those excluded from analysis due to excessive motion)

|  | White Ethnicity (England+ Wales) | White Ethnicity  (Current Study) | Black Ethnicity (England+ Wales) | Black Ethnicity  (Current Study) |
| --- | --- | --- | --- | --- |
| Age (years), median (IQR)^a^ | 42 (30) | 24.0(6.0) | 27(40) | 25.1 (11.2) |
| Gender, (%male) | 49.0% | 50% | 47.8% | 45% |
| Index of multiple deprivation decile (median, IQR)^b^ | 5 (5) | 4(4) | 8 (3) | 8(3) |

^a^ Census categories only available for five year brackets, so midpoints of brackets reported.

^b^ 1 is the least deprived and 10 is the most deprived decile

**Supplementary table 2:** Average age, gender, and indices of multiple deprivation for individuals in the current study and for the general population in England and Wales

|  |  | Black Ethnicity group  (white faces>black) | White ethnicity group  (black>white) | Between group contrast |
| --- | --- | --- | --- | --- |
| Within group | Right | T=2.4, p =0.015 | T=1.9, p = 0.036 |  |
|  | Left | T=0.2, p =0.42 | T=1.4, p=0.087 |  |
|  | Bilateral | T=1.3, p = 0.11 | T=1.8, p=0.044 |  |
| Between group (Black faces> fixation cross) | Right |  |  | (W>B) t = 1.8, p=0.040 |
|  | Left |  |  | T=0.83, p=0.21 |
|  | Bilateral |  |  | T = 1.5, p = 0.076 |
| Between group (White faces> fixation cross) | Right |  |  | (B>W) t =1.8, p= 0.038 |
|  | Left |  |  | T=0.98, p=0.17 |
|  | Bilateral |  |  | T=1.53, p = 0.068 |
| Group x Task | Right |  |  | F=7.85, P=0.008 |
|  | Left |  |  | F=1.84, p=0.46 |
|  | Bilateral |  |  | F=5.29, p=0.028 |

Amygdala ROIs were taken from the Automated Anataomical Labelling ROI library (a bilateral amygdala ROI was constructed by combining left and right amygdala masks)

**Supplementary table 3:** Imaging results for amygdala ROIs

*Neighbourhood variables within black ethnic minority group*

Normally distributed variables: amygdala response to white faces, population density, index of segregation

Non-Normally distributed variables: own group ethnic density, Index of multiple deprivation ranking

Outliers**:** one individual had an excessively high value for Index of dissimilarity (>2.5 SD above the mean) and so was not included in analyses of this variable.

*Relationships between Neighborhood Variables in the Black Ethnicity group*

Within the black ethnic minority group own group ethnic density negatively correlated with population density (*r_s_* = -0.787, *p*<0.001), negatively correlated with degree of segregation (*r_s_* = -.809, *p*<0.001), and positively correlated with greater deprivation (*r_s_*=.495, p=0.026). Segregation positively correlated with population density (*r_p_* 0.765, *p*<0.001) (a summary of correlations with is shown in supp figures 1 and 2).

*Relationship between amygdala response and neighborhood variables within the black ethnic minority group*

Within the black ethnic minority group bilateral amygdala response to white faces was greater in individuals living in areas that were: of lower own group ethnicity (r_s_=-.514, p=0.035), more segregated (rp = 0.577, p=0.019, after removal of outlier identified by cook’s d:r_p_=0.704, p=0.003); more densely populated (r_p_=0.609, p=0.009), and less deprived (r_s_=0.514, p=0.035) (see figure 1).

Within the black ethnic minority group right amygdala response to white faces was greater in individuals living in areas that were: of lower own group ethnicity (r_s_=-.611, p=0.009), more segregated (r=.710, p=.002, after removal of outlier identified by cook’s d: r_p_=0.831, p<0.001); more densely populated (r_p_=0.627, p=0.002), and less deprived (r_s_=0.601, p=0.011) (see figures 2).

For the stepwise regression all participants with valid neuroimaging data were included. At step one of the analysis, own group ethnic density was entered into the regression equation and was significantly related to amygdala response (R=0.689, p=0.002). At step 2 of the analysis no other variables entered the equation: index of dissimilarity (r (partial correlation)= -.134, p=0.621), population density (r=0.204, p=0.449), deprivation ranking (r= 0.433, p=0.211).

Relationship between variables in the White Ethnicity Group

Within the white British ethnicity group population density negatively correlated with own group ethnic density (*r_s_*=-0.88, *p*<0.001), and positively correlated with greater deprivation (*r_s_* = 0.519, *p*=0.013). No significant correlations were observed between neighbourhood variables and right amygdala response to black faces.


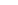

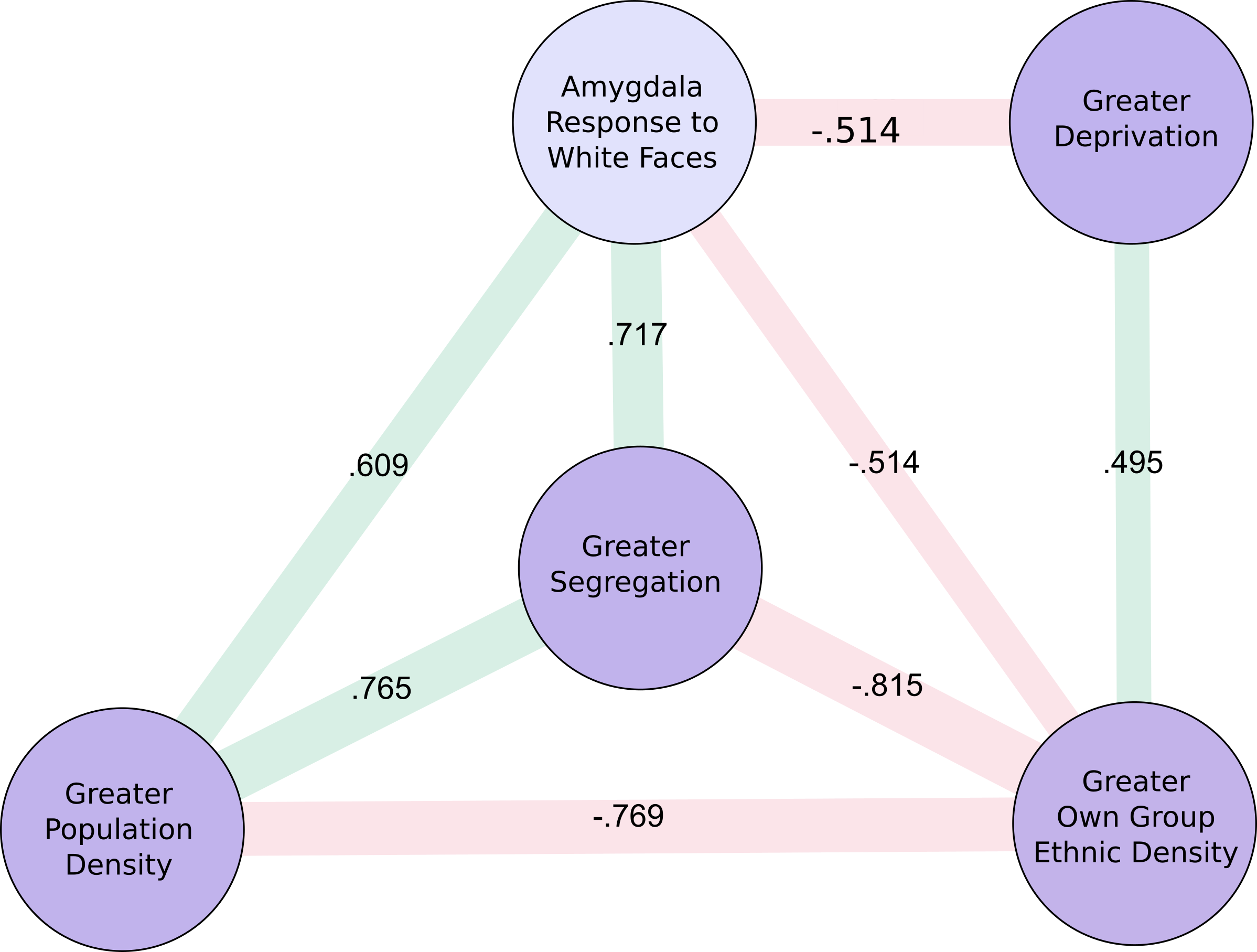


.704

**Supplementary Figure 1**: Significant correlations between neighbourhood variables and bilateral amygdala response within the black ethnic minority group. Green = direct correlation, Red = inverse correlation. Numbers = correlation coefficients.

**
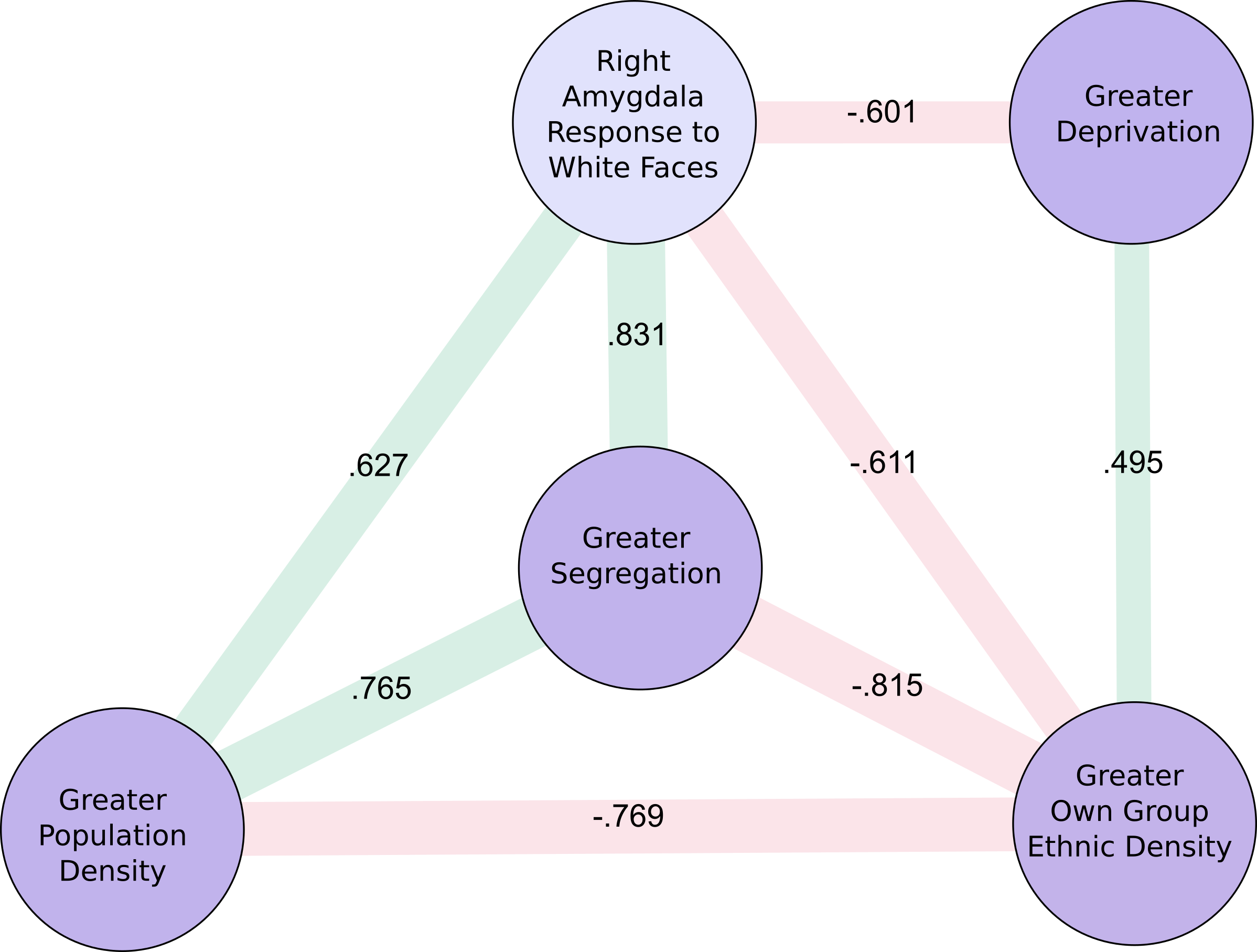
**

**Supplementary Figure 2**: Significant correlations between neighbourhood variables and right amygdala response within the black ethnic minority group. Green = direct correlation, Red = inverse correlation. Numbers = correlation coefficients.
